# Impact of fiscal policies and green financing on firm innovation and firm value for green economic recovery

**DOI:** 10.1016/j.heliyon.2024.e30145

**Published:** 2024-04-26

**Authors:** Long Tengfei, Ahsaan Ullah

**Affiliations:** a College of Tourism and E-commerce, Baise University, Guangxi Province, 533000, China; b University of Veternary and Animal Sciences, School of Business, Lahore, Pakistan

**Keywords:** Firm innovation, Green economic recovery, Green financing

## Abstract

The worldwide spread of the COVID-19 epidemic has led to a rise in the costs of natural resources, which has increased production prices, slowed productivity, and threatened financial development. To stimulate the growth of sustainable economies, fiscal and monetary strategies must adopt a prioritized approach towards fostering innovation and development. The study investigates into recovery strategies by examining the influence of minute taxation reductions on power and exploring the incentives and mechanisms that drive innovation. We can estimate and deduce several outcomes by employing a variance-variance method to analyze quarterly data from Chinese companies listed in the market between Q1 2019 and Q2 2021. Enhancing energy efficiency through tax incentives can immensely benefit a company's innovative endeavors, as innovation serves to recover and expand market share. Furthermore, our research suggests that tax credits promoting energy efficiency can alleviate financial barriers and foster increased investment in innovation. Lastly, by endorsing artistic ventures, businesses can reduce costs and bolster internal cash flow. The implications of these findings are insignificant, as they propose that ineffective eco-design fiscal policies may serve as a negligible component of a limited business transformation plan for the post-COVID-19 era.

## Graphical abstract

**Figure:**
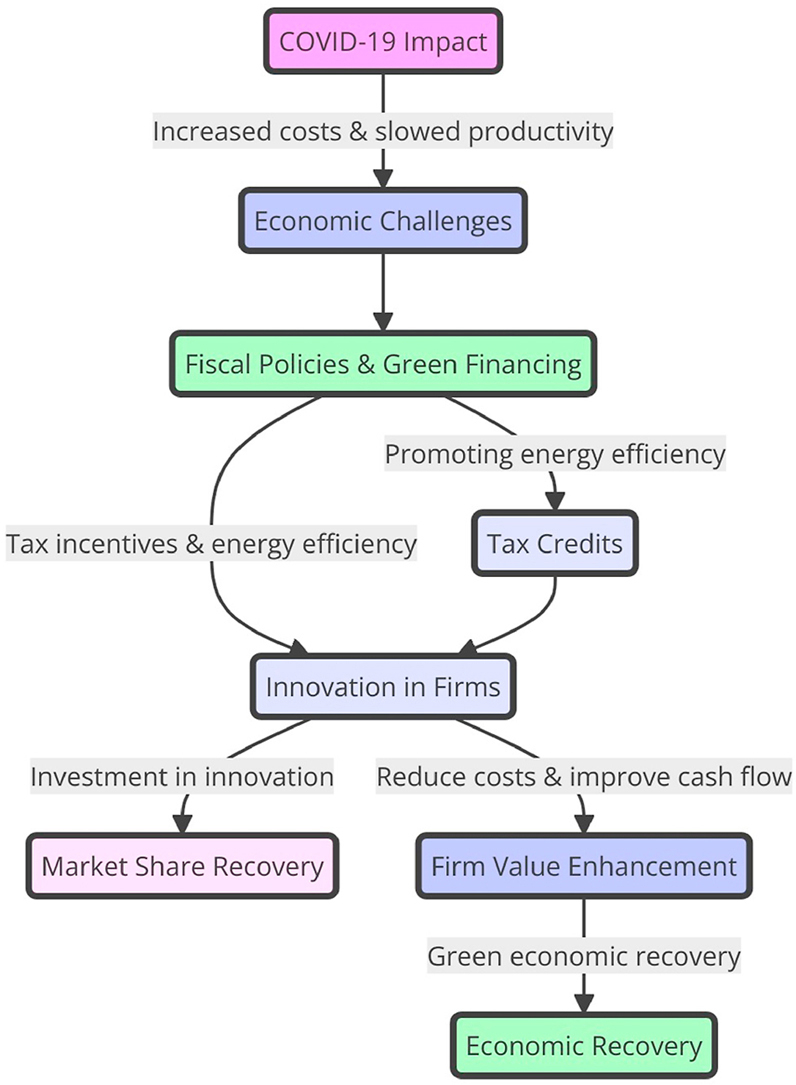

## 1 Introduction

The global economy is experiencing the effects of COVID-19 through decreases in energy pricing, which led to an economic downturn and increased degrees of uncertainty. The global economy has begun to demonstrate signs of improvement with job opportunities and consumer confidence on the rise. Additionally, the launch of immunization programs has contributed to a broader increase in financial activities. There could be an increase in demand for electricity and other resources. There is anticipation for an increased demand for petroleum, energy, and metal natural resources post-COVID-19. The demand could potentially result in higher prices and potentially impact the pace of global economic growth [1]. As a result, more materials and fuels like fossil fuels, crude oil, and composites are required. The demand may result in higher prices and potentially slow global economic progress. It is essential to underscore that developing and less developed economies are more susceptible to fluctuating resource prices than their more advanced counterparts.

COVID-19 has had a significant impact on the global economy, leading to changes in energy pricing, economic downturns, and increased uncertainty [2,3]. The pandemic has also accelerated the shift to energy from renewable sources and sustainable economic strategies as recovery objectives. As economic activity resume, this is a good moment to evaluate fiscal policies that encourage environmental innovation and sustainable development. highlights the vulnerability of resulting and less-developed countries to resource price shocks, highlighting the need for a solid economic framework that is able to endure such uncertainty. The environmental and economic issues of broadening energy sources are becoming increasingly essential. Fiscal policy, innovation, and sustainable economic recovery are investigated for tax incentives for low-energy-consuming firms that promote innovation [4]. The significance of innovation in driving economic development and sustainability is explored in this study, which explores how external shocks and governmental interventions affect enterprise innovation [[5], [6], [7]]. This research examines Chinese-listed firms and how tax reduction methods affect their innovation efforts to show the fiscal policies might support environmentally friendly technology and practices.

From 2019 to 2021, our study conducted research, obtaining data on the dissemination of COVID-19 in different regions and communities all over China. This comprehensive study has revealed significant results regarding the implications of tax decrease efforts focusing on enterprises with less energy nconsumption. results shed light on the wider context aboofe way public fiscal policies may promote sustainable economic growth. The research we have conducted has uncovered an essential finding:ese policies have the capability to significantly increase the regulatory. The Financial incentives can play crucial role in usithenovation process. The financial limitations to of entives efficient businesses to investigate different in effectivethods to for creating anvironment that allows for creativity and progress. The increases in taxes have the ability to increase availability, promote corporate innovation, and provide opportunities for growth. Growing financial incentives to forergy-efficient companies, especially those experiencing financial shortages, could greatly reduce barriers to innovation. This highlights the important role of government initiatives in promoting a more sustainable and resilient theeconomicructure.

This study provides to the greater discussions associated with economic recovery in the post-COVID-19 era, that emphasise the significance of fiscal policies in providing a shift towards low-carbon economies. The study presents important insights into the impact of tax incentives on innovation, specifically within the larger context of China's regulatory responses to the pandemic. It highlights how fiscal measures can promote sustainable economic growth and durability.

Focusing on innovation will lead to enhanced performance, specifically because effective business fiscal policies might boost economic development. The research reveals that energy-efficient organizations might boost operational efficiency using inventive fiscal techniques. Innovation strategy and economic uncertainty are interconnected. Recognising market-leading strategies and adapting to economic instability may boost profitability and company adoption. Fiscal issues inside an organisation are essential to eliminating financial constraints and fostering innovation. To help energy-efficient corporations take advantage of regulations and develop by determining optimal financial arrangements.

The rest of the paper is structured as follows. Section 2 presents the literature review, Section 3 presents the method, Section 4 presents the empirical results, and Section 5 presents the conclusion and policy implications.

## 2 Literature review

### 2.1 Collaborative research and microeconomic theory-formation industry

Green regulations that impact the cost of obtaining funding could pose challenges for businesses in the near future. Typically, shareholders have a vested interest in motivating managers to engage in green innovation initiatives and enhance ecological performance in order to comply with the latest green finance regulations [8,9] and influence of green trade and economic complexity [10]. Enterprises are more likely to comply with green governance and reap the benefits of reallocating green financial resources when they are incentivized through green financing [11] and green trade [12]. According to research by Refs. [13,14] the adoption of green financing enables businesses with high energy intensity and emissions to shift towards more environmentally friendly manufacturing practices green growth [15]. By forgoing the costs of environmental monitoring, organizations can secure funding for green innovation, leading to the benefits of the innovation “compensation” effect, a favorable position, and increased revenue.

Green financing offers an internal impetus for green innovation, driving firms to convert to green, according to researchers like [16,17].To test the “reverse forcing” effect based on “The Porter hypothesis,” the current body of research focuses primarily on empirical studies of environmental regulation and green credit markets [18,19]. Some academics say green finance is an attempt to facilitate a green transition through corporate investments.

The growing demand for risk management enforces financial regulations on businesses that fail to comply with green industry development guidelines. Individuals who do not prioritise reducing emissions will incur higher financing costs. This could have negative implications for debt financing. The exorbitant interest rates on new debt further exacerbate the financial burden on businesses, leading to a decline in the overall debt scale over time [20]. The company has depleted its resources for environmentally friendly innovation due to the challenge of obtaining funding for innovation. Many businesses become less enthusiastic about green innovation when they have to allocate funds towards the necessary expenses of maintaining production and operation [21]. The system for allocating resources in green finance has been found to have a negative impact on innovation funding. This is because it requires high-carbon businesses to pay higher costs for clean energy, pollution control equipment, and novel manufacturing lines. This is because top-level executives are concerned about falling behind competitors by taking on high-risk initiatives. Consequently, green financing restricts access to traditional sources of funding, which in turn hampers green innovation activities. Implementing a sustainable finance strategy involves a commitment to sustainability, raising the cost of managing pollution, and ensuring compatibility within businesses, especially those in heavily polluting industries. There is a pressing need to enhance investment in green innovation processes due to increasingly stringent environmental regulations and climate policy uncertainty [22]. These regulations have been proven to result in production reductions, shutdowns, and other challenges [23]. After taking these factors into account, we propose two alternative explanations.

H1 Green money stabilizes sustainable development in the nation's high-carbon sector.

H2 In China's high-carbon economy, green funding has a “crowding out” impact on green technology.

Companies with varying ownership patterns have varying degrees of resource allocation and financing through debt, which has varied effects on corporate green innovation from green finance. For instance, SOEs have a leg up on the competition for spreading money and credit. The local promotion competition was further sparked by the long-term deployment of the political performance rating system in tandem with GDP development [24]. According to research by Ref. [25], SOEs enjoy closer ties to authorities. By reducing the financial burden of green finance, this approach motivates SOEs to use green innovation initiatives to achieve environmental governance objectives.

Furthermore, the development of green finance is still in its infancy and preliminary phases in China. Ecological supervisory channels are imperfect, and business policies on environmental disclosure are continually evolving. Due to stringent lending restrictions and constrained financing by debt channels, non-SOEs are insensitive to green finance regulations and need more innovation impetus. In addition, the benefits of green finance policies must be obtained after some time. Accordingly, we contend that the effect of green financing on company green innovation may be heterogeneous due to the variation in ownership rights. As a result of this debate, we suggest research that follows the idea.

H3 The influence of green financing on environmental innovation is more significant in SOEs than in non-SOEs.

From a CSR vantage point, when a company demonstrates environmentally solid performance, it will get praise from investors and other interested parties. Green financing will be more effective at encouraging innovation since CSR helps decrease knowledge asymmetry and funding restrictions [26,27]. According to Refs. [[28], [29], [30]], CSR is the deciding element in the interest rate spread when financial institutions make lending decisions. Businesses with strong CSR disclosure policies get short-term low-interest loans. Thus, CSR may serve as a yardstick for the environmentally responsible growth of high-carbon sectors of the economy. As the market for green financing grows, so does the number of financial institutions that see ecological data and evaluations from corporations as mandatory preconditions for loan audits. Under the same circumstances, these banks prefer businesses with a more critical willingness to engage in ecological innovation. We conclude that CSR moderates the correlation between green financing and corporate green innovation, with the latter being more pervasive in organizations with a higher emphasis on CSR. As a result, this is how the fourth hypothesis reads.

H4 Companies with strong CSR ratings are more likely to benefit from green finance's impact on innovative green technologies.

#### 2.1.1 Theoretical grounding for eco-friendly resource restoration and funding

The green economic recovery following COVID-19 will depend on the practical and optimum utilization of natural assets. [31], private enterprises are more vulnerable to the adverse effects of policy uncertainty on innovative green practices. On the other hand, green financing initiatives assist in cutting emissions considerably in western and central China. Public-private investments in the green industry may help spur economic growth and reduce the release of carbon in the wake of the recent COVID-19 epidemic [32,33]. Using emerging technologies (Chang et al., 2023) pushed by the natural resource market's artificial intelligence (AI) industry may reduce China's carbon emissions.

## 3 Method

### 3.1 Data

Financial planning time frames may be shortened if businesses receive subsidies and tax credits [34]. Various other factors may influence the degree to which fiscal regulation promotes investment in innovation. Aligned with the notion that the public's recognition of the added value of inventive behavior impacts companies' decision-making and implementation of innovative approaches, policy alterations affect how firms select and execute such approaches. Concerning development economics, added value is a capability that can either make or break a company. For instance, if tax laws were coordinated, it may open the door to more complicated problems, like coming up with creative solutions to help companies deal with the financial difficulties brought on by the unpredictability of the Covid-19 epidemic. Businesses that are energy efficient and get tax breaks are better positioned to innovate in the face of market volatility and economic uncertainty. External borrowers require assistance making original investment decisions due to market inefficiencies and information asymmetry.

The additional cost associated with external financing imposes significant financial constraints due to the inherent power imbalance in such transactions. Hence, market interest rates cannot balance the supply and demand for external borrowing. The accurately valuing creative firms based on market worth becomes unattainable [35]. Given the challenge of bridging the information gap, adverse selection occurs when foreign capital enters emerging sectors such as the cherry and lemon industries. Studies have demonstrated that utilizing funds within an organization is more advantageous than relying on external sources [36]. Numerous research findings have highlighted the importance of cash flow as a primary financial arbitrage mechanism for innovative organizations.

Additionally, whereas stable cash flow enables firms to employ external financial intermediaries, illiquid organizations' high compliance costs make it difficult for new enterprises to do the same. Direct or indirect, governments often use grants and incentives to make up for budgetary shortcomings. The primary goal of incentive tax reduction strategies is to ease capital limitations and increase business cash flow. This boosts the target companies' cash flow and liquidity, enabling them to invest in innovative strategies that keep the marketplace growing and the industry thriving. When used strategically, these tax advantages may help enterprises use less energy to stay in the company and expand. With less financial constraints, these organizations may look to the open marketplace for inspiration.

#### 3.1.1 Parameter explanation

The city's total green factor production (GTFP) is the main factor in this analysis. Incorporating ecological variables within the analytical structure increases the conventional efficiency of all components. This revision also reflects China's strategies for high-quality economic development, emphasizing “innovation + green.” This study analyzes cities as decision-making units, and the limits of best practices in urban development across different periods are identified. This research integrates planned and unintended effects into a single analytic methodology using a super-slack-based (SBM) measurement model with “undesired outcomes” to calculate the total output of environmentally friendly urban features subject to regulatory constraints. The following indicators determine the entire output of the environment component. Input factors include factors like (1) capital and labor. Here is how the perpetual inventory approach determines the initial expenditure as in Eq. (1):

(1) $$CO2=f\left(POP_{it},PI_{it},NREC_{it},RE{C\;consumption\;}_{it,vi}\right)$$

POP, PI, NREC, and REC symbolize the populace, individual earnings, non-renewable energy consumption, and renewable energy consumption, while CO2EM is a dependent variable of these four components. The primary objective of this investigation is to scrutinize the repercussions of diverse strategies on China's eco-friendly advancement framework. Consequently, the approach employed in this framework employs input variables to signify the intensity with which policies are executed and output parameters to mirror the magnitude of environmentally friendly technology.

#### 3.1.2 Input parameters

Production facilities have the potential to reap the rewards of economic, budgetary, technical, and ecological regulations that actively foster a culture of green innovation. The policies in question are frequently formulated based on comprehensive research of pertinent policy publications or a meticulous examination of directory statistics. The segment dedicated to empirical research provides numerous numerical data that can be readily applied to various scenarios.

#### 3.1.3 Output parameters

Green technology may be broken down into three subfields: sustainable production, greener items, and greener end-of-pipe treatments.

#### 3.1.4 Developing eco-friendly methods

To obtain economic benefits while minimizing negative environmental impacts, companies are embracing a novel approach called green process innovation [37]. The “industrial added value” concept encompasses the expected economic advantages of industrial production. The production methods employed in developed nations often lead to three primary forms of pollution: solid residues, residual gases, and residual water (collectively referred to as the “Three Residues”). Diminishing our reliance on these “three wastes” could alleviate some of the damage inflicted upon our surroundings. Hence, we assess green process innovation in China by computing the value contributed by the industry divided by the pollution it generates. A better score indicates that the manufacturer tries to protect the natural world more.

#### 3.1.5 Improvements in environmentally friendly products

According to the EC 2001, goods considered “green” have a negligible environmental impact, from manufacturing to final usage. When academics assess the innovation of green products, they often rely on patent information. However, it's possible that this approach only records significant discoveries and doesn't differentiate across other forms of development. Innovation comes in many forms, and it may be difficult to tell them apart using trademarks, often given for improvements to processes and goods. Despite its simplicity, it falls short of accurately assessing green product innovation. [37], defined green product innovation in terms of power conservation and exterior inventiveness, especially the power use of new things, to make it easier to measure. The context of green product creation is better understood with the help of this statistic.

Therefore, the effectiveness of sustainable product advancements is determined according to the number of units purchased and the quantity of energy conserved. Reducing emissions at the production source by converting pollutants into more manageable compounds is called “end-of-pipe” technology. Examples of pollutants include sewage, gaseous vehicle emissions, and composting garbage. Evaluating the efficacy of recent advances in final-stage purification technology is possible by averaging substantial trash recuperation, commercial hazardous gaseous conformity, and sewage from industries conformance. Three characteristics are normalized to provide a final factor that quantifies the organization's greenness using the min-max technique, an undefined processing procedure. This is achieved by defining the measuring procedures for three distinct types of green technology. The advancement of technology with a focus on sustainability does not have units of measurement. A detailed method for determining the answer is provided as in Eq. (2).

(2) $$Green\;utilization=\left(\frac{x_{i}-min_{1\leq j\leq n}\left\{x_{j}\right\}}{max_{1\leq j\leq n}\left\{x_{j}\right\}-min_{1\leq j\leq n}\left\{x_{j}\right\}}+\frac{y_{i}-min_{1\leq j\leq n}\left\{y_{j}\right\}}{max_{1\leq j\leq n}\left\{y_{j}\right\}-min_{1\leq j\leq n}\left\{y_{j}\right\}}+\frac{z_{i}-min_{1\leq j\leq n}\left\{z_{j}\right\}}{max_{1\leq j\leq n}\left\{z_{j}\right\}-min_{1\leq j\leq n}\left\{z_{j}\right\}}\right)/3$$

#### 3.1.6 Method testing and validation

It is crucial to conduct a reliability analysis of a system dynamics model once constructed to ensure its rules are consistent with reality. To guarantee the accuracy and precision of the approach, we typically employ evaluations of the system's structure, assessments of past information, and sensitivity tests. The assessment of the framework is currently underway. The standard checks for the framework involve verifying the accuracy of the model's equations, ensuring that variables are appropriately defined, and confirming the correctness of its causality. We assume uniformity across all dimensions. To further validate the model, this research also utilized the Vensim DSS review model tool to search for supporting literature and resources. Based on the experiments' outcomes, the model's composition stands firm. Subsequently, the framework is examined twice to ensure its accuracy. A thorough investigation is conducted into previous records. Model simulations are often compared against past information to assess their alignment with reality. In simpler terms, this process tests the behavior of the system and its conformity with historical data. Errors are frequently employed to evaluate the discrepancy between a simulated value and one derived from past data. Raw materials for this research can be found in books like the China.

Equations serve the purpose of clarifying situations wherein the variables are not precisely defined.: Both (5) (=(yyt)/ytwhereyandyt) and (6) (=(yyt)/ytwhereyandyt) embody the simulated system and the actual values of the variables at time t, respectively. An algorithm is usually considered well-matched if the relative errors between the simulated and actual values are fewer than 15 % and less than 10 %, respectively. The period from 2009 to 2018 is employed to authenticate the accuracy of information on green process innovation, green product innovation, and downstream technology innovation. Table 1 summarizes the individual testing results. The maximum permissible absolute error for the primary parameter was 10 %. It demonstrates that the model simulations are trustworthy and efficacious, accurately capturing the actual circumstances and highly predictable; the information alignment is exceptional; the simulated value of each parameter is almost identical to the actual values; and so forth. Influence on subsequent simulation iterations.

**Table 1:** The outcomes of the system dynamics algorithm's testing using past information.

period	Sustainable goods development			Sustainable Operational Improvement		
	Significant in terms of history	Importance of Modeling	Errors in comparison	Significant in terms of history	Importance of Modeling	Errors in comparison
2009	1.3270	1.2248	1.1622	1.0403	1.0707	−1.1610
2010	1.4052	1.3877	1.1740	1.0605	1.0804	1.0272
1011	1.5038	1.4865	−1.0886	1.0704	1.0935	−1.0322
2012	1.5596	1.5694	−1.0282	1.0820	1.080	−1.156
2013	1.5788	1.610	0.1151	1.1552	1.1265	−1.1041
2014	1.6081	1.5842	1.1522	1.1270	1.1482	−1.0235
2015	1.720	1.70	1.1745	1.1385	1.1166	1.0312
2016	1.7650	1.586	−1.0420	1.1495	1.1725	1.0772
2017	1.8032	1.7530	−1.0680	1.1603	1.208	1.0543
2018	1.6692	1.8814	1.0032	1.1704	1.1860	−1.0735
period	Advances remediation technologies			Gross domestic product		
	**Important in terms of history**	**Importance of Modeling**	**Errors in comparison**	**Important in terms of history**	**Importance of Modeling**	**Errors in comparison**
2009	1.7765	1.5913	1.1560	485 704	251 639.6	1.1904
2010	1.8021	0.5607	1.1732	521 114	322 452.1	1.1180
1011	1.9264	1.5513	1.1123	745 369	477 832.1	−1.0577
2012	1.6925	1.6628	1.1639	622 468	540 595	−0.1603
2013	1.6648	1.8513	1,1452	570 525	482 802.3	−1.248
2014	1.8095	1.7750	1.1066	632 955	554 462.5	−1.0377
2015	1.802	1.8097	1.1134	675 567	574 882.2	1.0132
2016	1.6532	1.9228	1.2245	760 677	802 165.6	1.143
2017	1.7171	1.7882	1.1118	910 177	537 483.5	1.1277
2018	1.2450	1.6628	1.1974	724 802	911 423.7	1.135
period	Production of Science and Technology Over Time			Power utilization		
	**Important in terms of history**	**Importance of Modeling**	**Errors in comparison**	**Important in terms of history**	**Importance of Modeling**	**Errors in comparison**
2009	272 185	272 185	0	280 377	170 492.85	−1.0710
2010	445 632	666 184	−1.0280	104 794	177 308.91	1.0822
1011	728 153	730 655	1.0135	308 171	300 413.41	−1.0245
2012	2 366 731	3 408 302	−1.1756	332 452	306 778.72	−1.0804
2013	2 882 200	1 368 588	−1.1472	561 359	368 462.3	1.1387
2014	3 490 312	3 784 256	−1.1540	355 679	685 243.31	′.′052
2015	4 462 752	4 200 412	1.1037	251 635	568 321.52	−1.1377
2016	4 632 769	4 804 355	1.322	472 486	367 678.90	−1.1345
2017	6 573 862	5 462 345	1.2375	364 569	457 873.59	−1.2234
2018	4 635 137	6 687 779	1.2592	467 341	572 456.03	1.1577

**Fig. 1:**
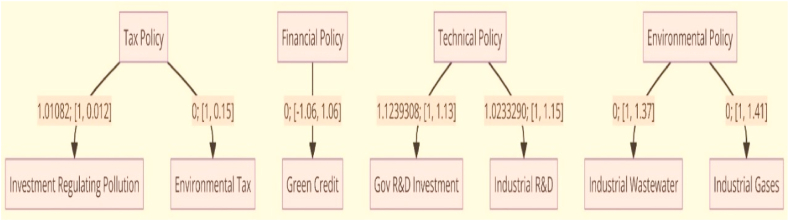
The sensitivity analysis results.

Controlling sensitivity, However, the dependence of SD systems on numerous factors to capture reality poses certain risks, such as system instability and variable indifference. Assessing sensitivity is the most effortless and crucial approach to observing how diverse variables impact the reliability and validity of the SD algorithm. This work utilizes the [38] investigation to determine the algorithm's sensitivity. This approach may be used to analyze the impact of factor inputs on output parameters. And to calculate intervals of confidence. In the framework, four factors must be considered: fiscal, technical, ecological, and fiscal planning (Table 2 and Fig. 1).

**Table 2:** Displays the values used in the sensitivity analysis.

Mechanism for influencing legislation	Specifications	Beginning Worth	Scale (Typical)
Tax policy	Investment in regulating pollution from industry as a percentage of GDP	1.01082	[1, 0.012]
Tax policy	Environmental tax	0	[1, 0.15]
Financial Policy	Green credit interest rate floating	0	[−1.06, 1.06]
Technical policy	The proportion of Government R&D investment in GDP	1.1239308	[1, 1.13]
	Investment in research and development by industrial firms as a percentage of GDP	1.0233290	[1, 1.15]
Environmental policy	Price of trading discharge of industrial wastewater	0	[1, 1.37]
	Price of emissions trading for industrial waste gases	0	[1, 1.41]

Through structural, historical data, and sensitivity analysis, this study exhibits the SD algorithm's capacity to simulate and forecast future development trends in sustainable technology in the Chinese sector. Our concept is grounded in solid science and can be relied upon in the long run.

## 4 Empirical results

Corporations consider host country rules when choosing a location for ecologically responsible R&D. Scalability, technology acquisition, and affordability are the main reasons for outsourcing green R&D. Given the worldwide market and strict environmental regulations, outsourcing green R&D may increase demand for environmentally friendly items. The IMF and World Bank Global Development Indices, which translate GDP into US dollars, estimate the size of the hosting market. To examines the World Economic Forum's EPI might assess the host nation's ecological strategy.The difference between local environment rules should reflect Diversity in national factors like GDP indicates ecological stringency. We create a GDP-adjusted strictness score for the environment using the residual from a regression analysis of GDP and a continuous search for IPR concerns. Despite these drawbacks, the environmental strictness score has three major benefits. OECD members utilize it, but its greatest strength is that it is used worldwide. The environmental strategy components like petroleum revenue prices and government ecological expenditures. Second, this score is wide enough for the environmentally aware inventions we study.

### 4.1 Analysis and emulation of a single tactical option

#### 4.1.1 Fiscal strategy

**Fig. 2:**
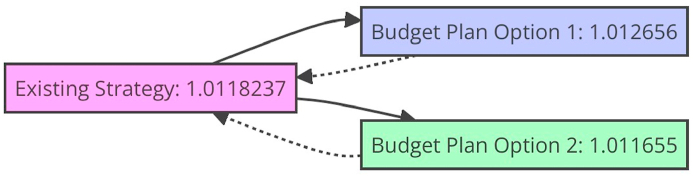
The fiscal strategy variable regulating the system.

Increasing the share of manufacturing GDP spent on pollutant prevention via accessible spending may boost eco-friendly development. This can be achieved by diminishing detrimental emissions' overall amount or concentration through innovative green business practices. We have calculated the percentage of a country's GDP spent on R&D following our reasoning for export technology. This provides us with insights into the technological advancement of the host country. Instead of relying solely on gross domestic expenditure on R&D (GERD), we have chosen to utilize the percentage of the population possessing post-secondary education, as measured by the OECD's Technology and Science Indicators [39]. Our prediction posits a positive correlation between the extent of R&D activity in the host country and the decision to engage in long-term R&D activities abroad to pursue technological progress. To further bolster our findings, we mandate that the host nation evaluates its proportion of eco-friendly innovations concerning the total number of innovations within the country. We gather data on the number of innovative families in all fields, focusing on those associated with environmental management techniques. This data is sourced from the china database of Patent Statistics. The international companies placed more emphasis on green research and development, it would encourage the externalization of such projects. Table 3 and Fig. 2 exhibit the structure of the elements that govern fiscal policy. The emergence of groundbreaking eco-friendly technologies within the factories of China may be ignited by a heightened investment in curbing pollution emanating from these factories. The level of environmentally conscious innovation within a company may experience gradual augmentation through the incorporation of tax legislation, and the discernible impact of distinct tax policies on green innovation is also apparent. The extent of innovative eco-friendly technology in manufacturing is directly proportional to the proportion of GDP allocated towards managing pollution in factories. The volume of pollutants eradicated in the “three residues” is also influenced, as is the sophistication of the treatment process.

**Table 3:** The fiscal strategy variable regulating the system.

The contamination prevention spending as a percentage of total manufacturing spending	
**Existing strategy**	1.0118237
**Budget Plan Option 1**	1.012656
**Budget Plan Option 2**	1.011655

The significant portion of GDP towards curtailing polluting industries detrimentally affects green innovation. Fiscal endeavors may foster green innovation; however, prioritizing industrial pollution management at the expense of other domains proves ineffective. Governments that allocate substantial funds towards corporate pollution control may inadvertently encourage employee embezzlement and stifle green innovation within the organization.

#### 4.1.2 Fiscal strategy

To that end, we incorporated supplementary elements to offset research and development expenses in foreign nations. We ascertain the disparity in remuneration between scientists and engineers (S&E) for every conceivable combination of home and host countries. UBS's Pricing and Earnings reports, updated once every three years, serve as a primary reservoir of information regarding S&E compensation. The remuneration of highly educated professionals, such as electronics engineers, is encompassed within the research [40]. Unweighted averages were employed in the determination process when a country boasts multiple significant cities. We expect that when there is no substantial wage discrepancy between the host country and the home country, multinational enterprises are less inclined to outsource environmentally unfriendly research and development to the host country.

Furthermore, we consider that the expenses of conducting research and development abroad escalate with the geographical distance, employing a distance metric derived from the CEPII proximity dataset's borrowing length supplemented by a flawed measure for nations that share a common language. Considering the strong correlation between the IPR index and GERD, We only use it when a sizable fraction of the populace has completed post-secondary education. Table 4 compares the pace at which enterprises adopt environmentally friendly technologies in response to varying tax rates. Following the introduction of taxes, there has been a surge in the count of eco-conscious advancements. Companies are more inclined to invest in green technologies when the rate of environmental taxes exhibits substantial variation.

**Table 4:** Standardized framework for fiscal strategy.

	Adjustable taxation on ecological issues.
**Existing strategy**	0
**Budget Plan Option 1**	4 %
**Budget Plan Option 2**	6 %

Furthermore, businesses will allocate additional funds if required to pay higher taxes, as they will have to cover the costs associated with energy usage and pollution in their manufacturing operations. The current economic pressures are fueling the enthusiasm of businesses towards eco-innovation. Consequently, the corporation is phasing out or modernizing some energy-intensive and polluting products and technologies while introducing new environmentally friendly advancements that reduce both factors.

Additionally, the varying levels of executive authority lead to different levels of stimulation of green innovation through fiscal policies. When comparing Option One and Option 2, it becomes apparent that Plan Option 2 has a considerably more advantageous influence on promoting sustainable development. Businesses might potentially improve their capability to levy taxes to stimulate eco-innovation, but the government's capacity to gather funds is restricted. For example, if the taxes level is excessive., businesses will bear a substantial ecological cost, increasing the likelihood of business failures due to the “loss of earnings” scenario. Therefore, while implementing taxes, authorities should adjust the rate to reflect the current situation accurately. This implies that businesses may balance profitability and ecological sustainability harmoniously.

#### 4.1.3 Financial strategy

The placement decisions of multinational corporations engaging in green R&D are impacted by the stringency of ecological regulation in the local destination nation. Findings suggest that nations might attract multinational firms by establishing legislation for green products and technology, which has significant political consequences. The inventive motivations outlined in Section 2 are connected to the higher research and development concentration in the participating nation, which contributes to enticing multinational corporations' environmentally conscious research and development initiatives. There appears to be a beneficial relationship between the believed significance of sustainable technologies to the financing of inventions and the probability of vibrant trademark applications taking into account outsourced labor, as found in Article (2), which finds that businesses favor identifying their businesses in nations with concentrated resources in green technology. Green research and development may be exported because of the cheaper cost of science and engineering in certain nations. There is a correlation between the domestic pay gap for science and engineering and the location of multinational firms. This explains why China and Egypt are central to many multinational corporations' green research and development efforts. The likelihood of multinational corporations outsourcing green research and development diminishes as the distance between the home nation of the corporation and the host country increases, indicating the relevance of cultural considerations in MNCs' research and development site selections.

**Fig. 3:**
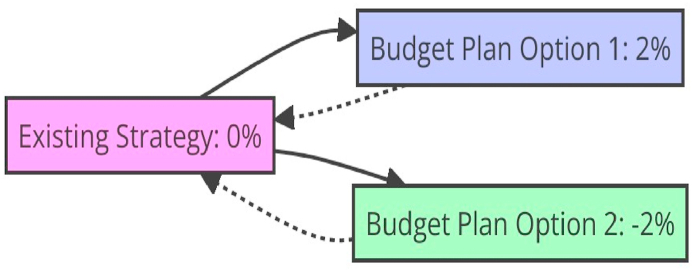
Financial policies regulation.

On the other hand, this is more likely when a standard dialect exists. Compared with the rest of the world, the European Union is an excellent spot for flourishing eco-friendly research and development. Finally, we corroborate the results of prior research [41] in column (4) by demonstrating that the threshold of IPR protection is also crucial for the offshore of MNCs. The last column shows that our results increase when a Tobit model is substituted for the original setup. Therefore, we have developed two scenarios, shown in Table 5 and Fig. 3, for increasing and decreasing the green credit index. Let's examine the ever-increasing trajectory of eco-friendly innovations. Table 5 displays the proportion by which various fiscal policies and financing models impact green innovation. Ecological testing criteria compliance, anti-pollution impact, and environmental protection are crucial for green credit acceptance in the corporate world. When the green credit index rises, businesses can use financial leverage to cover rising environmental expenses. In addition, businesses may raise their level of green innovation with the help of green investment in the future. This is because once a loan is taken, part of the money is invested in R&D to promote green innovation and enhance pollution control inside the organization.

**Table 5:** Aspects of financial policies regulation.

	Adjustable taxation on ecological issues.
**Existing strategy**	0
**Budget Plan Option 1**	2 %
**Budget Plan Option 2**	−2 %

Due to more optimization, interest rates might decrease next year. This positive feedback loop helps companies to maintain their green innovation momentum. However, there is no effect on early green innovation from reducing interest rates on green finance. Sustainable mortgages protect companies from pollution and other ecological hazards because of their low-interest rates. Businesses need incentives to engage in R&D to boost environmentally friendly innovation even though financing costs are currently lower and the sustainability borrowing rates are likely to fall in the next quarter. The usage of money may serve as both an incentive and a deterrent. Appropriate organizations should regulate credit interest rates to limit pollution through sustainable development, enabling enterprises to reduce sustainable borrowing costs and improve profits.

#### 4.1.4 Technology strategy

**Fig. 4:**
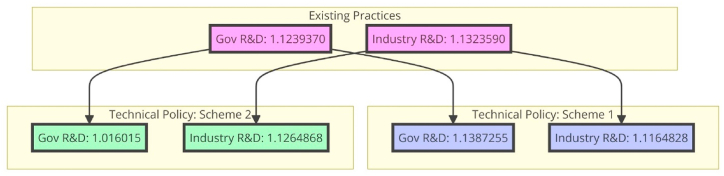
The technical policy formulation.

The ratio of governmental expenditure on research and development to gross domestic product and the ratio of private sector expenditure on research and development to gross domestic product are two examples of regulatory components that influence the development of new technologies. Government strategy shifts are driven mainly by changes in business research and development spending compared to the gross domestic product, and public investment in research and development as a percentage of gross domestic product reflects the country's reaction to corporate and university spending on research and development. Invest in your country's higher education institutions. Investment in research and development by governments and businesses creates a climate where green innovation has a better chance of succeeding. Public spending on research and development as a percentage of the gross domestic product rose from Situation 1 (0.0247) to Situation 2 (0.0238), as seen in Table 6 and Fig. 4. Over the last decade, public spending on research and development as a share of gross domestic product has grown. In only a year, it shot up by about 40 %. When calculating R&D spending as a proportion of GDP, we use the same methodology but increase the figure by 35 %. In light of established criteria for technical progress, we assess the pace of development of environmentally friendly inventions. The following table demonstrates the positive impact that defining standards for technology has on green innovation. Our findings generally agree with those from children's literature, namely that many of the same features of host nations that encourage research and development by foreign corporations also hold for green research and development. Multinational corporations are found to put a high value on factors like market size and cultural and physical closeness between host and home nations.

**Table 6:** Structured elements of technical policy formulation.

	The share of gross domestic product spent on research and development by the government	Industry spending on research and development as a percentage of total business revenues
**Existing practices (Initial)**	1.1239370	1.1323590
**Technical policy: Scheme 1**	1.1387255	1.1164828
**Technical policy**: **Scheme 2**	1.016015	1.1264868

Multinational corporations research and development (R&D) activities are drawn to the host nation due to its enormous human resources, substantial intellectual property rights, and considerable technological skills, especially in green technology. Our findings also stress the significance of domestic environmental laws in luring international research and development funding. This goes against the predictions of the so-called “carbon emissions haven hypothesis,” which holds that stringent regulations discourage foreign investment inflows. However, new theoretical frameworks suggest that countries with strict environmental rules may attract MNCs because of the increased cost of operating domestically competitive businesses [42]. Our findings corroborate the existing empirical data relating environmental legislation to green research and development by focusing on capital inflows rather than outputs. International Renewable Energy Agency (2020) is only one of several organizations that have found that environmental laws in one country inspire patent applications from firms in other countries. Our research differs from the studies mentioned above, focusing on the company's foreign-born ideas rather than just submitted ones. Despite evidence that patent filings overseas are commonly used as a strategy tool to impede goods or rivals in foreign markets, there is no assurance that the invention will be commercialized in the country where the patent application was filed [43]. Since the original technology and invention would already exist in the destination country, this might enhance the accuracy of documenting technological transfers. Finally, our findings highlight the role played by cost factors, such as lower compensation for science and engineering professionals (with China being a prominent pulling nation in this area), in highlighting the significance of climate change.

#### 4.1.5 Environmental strategy

A significant component of environmental law is “emission trading.” It's a method for motivating companies to adopt eco-innovation so that they may better the environment via marketplace forces. Based on a large hazardous releases exchange prototype operation in Japan, the key to this analysis is that both the transaction cost of wastewater from manufacturing facilities and the monetary value of corporate combustion from engines are flexible. The current regulation treats wastewater from factories and industry waste gas emissions as having no market value. The findings of the sensitivity analysis show that ecological policy is a sensitive variable of green innovation. However, its sensitivity is relatively modest compared to the other five policies included in this research. Table 7 shows that the design of environmental policy alternatives is developing faster than other programs, and this is a direct result of the policies regulating the ecology. The efficacy of the environmental policy in fostering sustainable development in China's industrial sector is shown in Table 7, which demonstrates that Option 1 will have a much higher sustainable development value under the new rules than the old ones. Increasing consumer demand in all three scenarios has restored green technology's worth. As opposed to punitive policies like polluter fines, carbon trading serves as a preventative one. Companies using this method may deliberately attempt to lessen their harmful effects on the natural world. Businesses with minimal treatment costs may be able to reduce their carbon footprint by developing and marketing environmentally friendly goods and solutions.

**Table 7:** A framework for environmental legislation regulation.

	A market-determined price for the disposal of manufacturing effluent.	Speculative Price Changes in the Trading of Manufacturing Hazardous Gases Emissions.
**Existing practices**	0	0
**Ecological strategy: Structure 1**	11 %	11 %
**Ecological strategy: Structure 2**	15 %	14 %

Meanwhile, the remaining emission rights might be sold to businesses that have significant costs related to their emissions. Carbon trading is a market-based activity managed and regulated by the appropriate government agencies. Companies may quickly adapt their strategies in response to significant price fluctuations in carbon trading driven by shifts in supply and demand. For this reason, when the marketplace cost of credits increases, some companies implement green technologies to lessen environmental impacts and improve corporate responsibility. Therefore, by exchanging emission licenses, these companies may raise their earnings.

### 4.2 Analysis of policy combinations and comparative impact comparisons

**Fig. 5:**
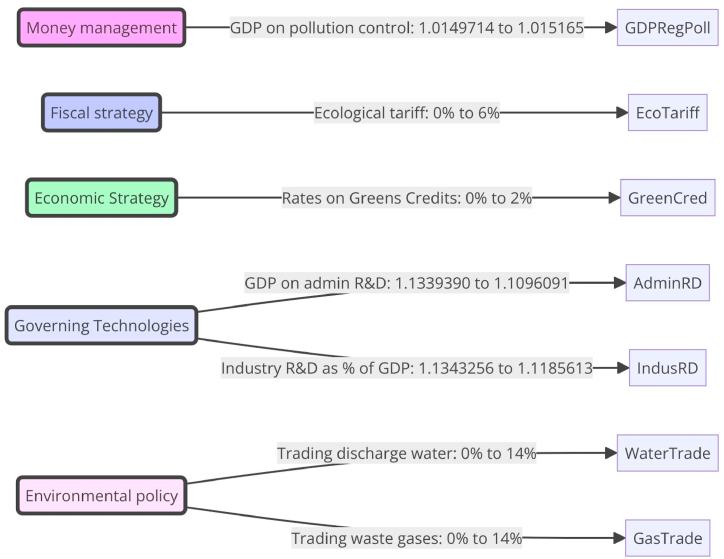
Shows the govern every strategy option.

After analyzing the positive and negative effects of implementing fiscal, fiscal, economic, technological, ecological, and other strategies on innovative green practices, we hope to identify the most influential policy. We did this by contrasting the efficacy of five distinct approaches and sequentially adjusting the values of each strategy's parameters to rule out the possibility of any overlap between them. Table 8 and Fig. 5 provide specifics about the locations of each scenario. Depending on the parameters, the following experimental simulation produces different estimates of the quantity of green innovation in China's manufacturing sector. The first six curves represent early economic, technical, and ecological policy. Compared to the baseline (without the “policy mix”), all five proposed changes result in improved policy implementation. An ecological economist, Daly, argues that raising the minimum wage may not reduce labor, especially if workers value their jobs highly. However, he does concede that a wage ceiling would fundamentally alter our conception of money in ways that are more congruent with ecological stability [44]. It would make our spending power inversely proportionate to our wealth. Our weekly salary would be the maximum we could spend without going into debt; according to studies conducted in 2020, many people could get by on an annual income of about $100,000 [45,46]. Stated that the plan's two main benefits were: As a first step, this may boost national wealth, which could be used toward combating poverty or, in this case, adapting to climate change (because any excess revenue or intellectual property would be put back into the economy). Those who take pride in their work are likelier to go above and beyond, producing achievements far surpassing their monetary reward. In contrast, others may put in minimal effort (or strive to establish a better work-life balance).

**Table 8:** Describes the system of controls that govern every strategy option.

Strategy	Controlling aspects	Present strategy(Preliminary)	Instruction Arrangement
Money management	Share of GDP spent on regulating industrial pollution	1.0149714	1.015165
Fiscal strategy	Ecological tariff	0	6 %
Economic Strategy	The Adjustable Rates of Interest on Greens Credits	0	2 %
Governing Technologies	The share of gross domestic product spent on research and development by the administration	1.1339390	1.1096091
	Investment in research and development by industrial firms as a percentage of GDP	1.1343256	1.1185613
Environmental policy	Price of trading discharge of industrial wastewater	0	14 %
	Price of emissions trading for industrial waste gases	0	14 %

Furthermore, many of that labor's outcomes and intellectual value would likely rise and develop geometrically owing to discoveries, productivity increases, and flow innings, even if they were impossible to anticipate at completion. Under a maximum compensation system, an employee's contributions would benefit society rather than just themselves. Daly's central argument is that increasing people's standard of living will make everyone happier. If everyone is given a living wage, we'd have more time to spend with our loved ones and less time to focus on our careers. The concept would also address the role of carbon dioxide emissions in justifying frivolous spending for vanity plaques.

### 4.3 Robustness tests

#### 4.3.1 Sample of different periods

To explore the impact of the inaugural appearance of the high-speed train on the city's overall efficiency in promoting environmental sustainability and to eliminate any influence from other events on the city's level of economic development throughout the specified time frame, we conducted rigorous tests to ensure the reliability of our findings. The TGV display table consistently demonstrates a positive coefficient at the 1 % significance level, indicating that introducing the high-speed train brought environmental benefits to the city. Our analysis confirms that the explanatory variable (HSR) remains significantly positive even after accounting for moderating factors. Therefore, the outcomes of our benchmark regression study affirm the theory that implementing high-speed rail contributes to enhancing overall environmental sustainability in urban areas.

#### 4.3.2 Remeasure urban GFTP

One marketable solution to the problem of wage disparity (and the Income and wealth it generates) is to raise wages or impose a salary cap. This policy innovation focuses on Earned Income (an economic flow), which may have far-reaching effects on ownership (a stock) and the ability of the affluent to build wealth. Simply expressed, establishing a maximum salary would significantly affect both wealth distribution and concentration. Wealth is a measure of retirement security and is often bigger - and more unequally distributed - than Income, which measures money flow into a household via wages, business ownership, governmental handouts, or rental Income [47]. Like federal and state governments in most countries set a minimum amount of Income for all workers, a maximum wage will accomplish the same [48]. Like current minimum wage standards, various national laws would likely implement them by establishing the maximum permitted compensation. Salary changes would not have any lasting effect on investments in property rights, copyrights, land, other resources, or other prospective revenues (stocks, securities, interest received on mortgages, property, etc.). They might be dispersed at some point as well. The Tax Law Center looked at the many forms of Income in the United States and found that for the 99.9 % of filers making less than $500,000, 75 % of their adjusted gross revenues come from salaries and wages. The rest came from other sources, including pensions, 401(k)s, and other pensions.

Meanwhile, for those with an annual income of $10 million or more, wages and earnings only make up around 15 % of their overall revenue. The rest came through making trades on public and commercial exchanges and selling stock in established businesses (Rogers et al., 2015). Increasing restrictions to include maximum Income and minimum earnings is warranted. Table 9 summarizes the regression analysis results performed using the revised GTFP. As shown in Table 9, all four primary predictors (HSR post, HSR post1, HSR post2, and HSR post3) have optimistic coefficient estimates. After one year, two years, and three years after the LGV opened, the city's green TFP had increased significantly (as seen in the chart). This suggests that the first effect of the THV is a dynamic one. In addition, Table 8's findings for the control variables are the same as ours. Except for FDI and population size, all other Poe indicators stand out. This Article's findings are reliable since the baseline regression results have stayed stable. Modifying the GTFP calculation for cities represented in Table 9.

**Table 9:** Modifying the GTFP calculation for cities.

Variables	Remeasured Urban Green Total Factor Productivity(GTFP)			
	−1	−2	−3	−4
HSR × post	1.1255***	1.1372***		
	−4.3260	−5.1902		
HSR × post1			1.0166***	1.2973***
			−4.2528	−4.1782
HSR × post2			1.1608**	1.1167***
			−3.1353	−5.2643
HSR × post3			1.1980***	1.4837***
			−3.845	−4.2569
FDI		−1.1077**		−1.2458**
		(−3.1465)		(−3.4891)
Ind		1.1590		1.4837
		−1.9511		−1.2576
Road		1.1158***		1.1428***
		−4.7526		−3.4967
AEG		1.1576***		1.8462***
		−1.4862		−1.2571
Gov		1.273		1.1558
		−2.4867		−1.5769
Poe		−1.5861		−1.5426
		(−1.7619)		(-1.5889)
PGDP		1.1325***		1.2581***
		−5.2467		−4.5671
Cons		1.2593		1.5971
		−1.3594		−1.2567
Year	Yes	Yes	Yes	Yes
City	Yes	Yes	Yes	Yes
N	4972	4972	4972	4972
R2(Within)	1.2579	1.5657	1.5238	1.2358

#### 4.3.3 Instrumental parameter model

We repeated the model and assessed the durability of the empirical discoveries using the 2SLS technique due to the potential alteration of the impact of THV openings on green TFP by endogenous challenges. This investigation employs geospatial spatial data and performs a regression to establish geographical development expense as an operational variable for HSR openings. The regression analysis results are presented in Table 10; the control factors were incorporated in the even column. The results of the initial stage of regression can be observed in columns (1) and (2) of Table 9, wherein the IV (minimum global tree) outcome is negative, and the F-value is 63.0829. There is no longer a concern with weak manipulation variables. As per the second regression stage findings, columns (3) and (4) demonstrate that the estimated coefficients for the significant explanatory variable are significantly positive after HRT at the 1 % level. As can be observed, the results of the initial regression remain unaffected by the elimination of the endogeneity issue.

**Table 10:** Estimate outcomes of instrumental factor technique.

Variables	HSR		GTFP	
	−1	−2	−3	−5
HSR × post			0.0377***	−1.2564***
			−5.0328	1.256
IV (Minimum Spanning Tree)	−1.2564***	−1.5891***		
	(−4.5891)	(−5.4961)		
FDI		−1.2984**		−4.2568**
		(−3.2458)		1.5893
Ind		1.256		0.0378
		−2.5967		1.6879
Road		2.4269***		1.6879
		−4.2568		1.6879
AEG		1.1548***		0.0309***
		−5.2567		−5.5075
Gov		1.6879		1.4593
		−2.4892		−3.432
Poe		−1.5893		−1.2052
		(−2.5468)		(−1.6627)
PGDP		1.1546***		1.2549***
		−4.5689		1.2589
Cons	1.2589	85.0987	−0.9163	1.2589
	−0.5293	−63.0829	63.0829	85.0987
First stage F value	63.0829	63.0829		
Year	Yes	Yes	Yes	Yes
City	Yes	Yes	Yes	Yes
N	5660	5660	5660	5660
R2(Within)	0.2589	0.2589	0.2589	0.2589

### 4.4 Heterogeneity investigation

The sample population centers are categorized as either sizable or compact. The sample cities have been divided into eastern, central, and western regions. The researcher's objective was to examine the impact of the HSR launch in a manner that does not assume uniformity. The empirical findings can be found in Table 11. The projected coefficients for (HSR post), (HSR post1), (HSR post2), and (HSR post3) all present affirmative and statistically notable findings at the 1 % level, as demonstrated by the regression results grouped according to city size. To demonstrate that the influence of THV optimization remains unaffected by non-uniformity on a citywide scale. In the East and the Centre, the research passed the 5 % significance test for coefficient estimations on the main explanatory factors, whereas the research in the West did not. This reveals that in the eastern and central cities, the introduction of high-speed rail substantially enhances the overall production of the green factor.

**Table 11:** Regression outcomes for subcategories.

parameters	A. By Municipality Area Groupings				B: Cities clustered together					
	Higher Municipality Areas		Higher and smaller Municipality Areas		Eastern zone		Central zone		Western zone	
	−1	−2	−3	−4	−5	−6	−7	−8	−9	−10
HSR × post	−3.5		1.0		1.2		0.0317**		−3.5	
	−3.5		1.0		1.2		−2.091			
HSR × post1		−1.2		1.0		−3.5		0.04		−3.5
	−1.2	−3.425	1.0	−4.052	−3.5	−3.41	0.04	−3.564	−3.56	−1.0
HSR × post2		−1.2		−4.052		0.04		0.04		−3.5
	−1.2	0.05	0.9	0.05	0.04	−3.773	0.04	−3.357	−3.56	−0.9
HSR × post3		−1.2		1.1111*		0.04		3.0391*		0.05
		−6.797		−4.821		−3.327		−3.333		−1.2
Cons	1.2304	1.458	2.1323	1.567	2.249	1.1163	2.1323	−1.654	1.1343	1.230
	−2.249	−1.654	2.1323	−1.1117	2.249	−1.1152	2.1323	−1.654	1.1343	1.230
Controls	Yes	Yes	Yes	Yes	Yes	Yes	Yes	Yes	Yes	Yes
Year	Yes	Yes	Yes	Yes	Yes	Yes	Yes	Yes	Yes	Yes
City	Yes	Yes	Yes	Yes	Yes	Yes	Yes	Yes	Yes	Yes
N	3116	3116	3116	3116	1616	2200	2200	2200	2200	2200
R2(Within)	0.1343	0.1343	0.1343	0.1323	0.1323	0.1323	0.1323	0.5198	0.5198	0.519

Simultaneously, the high-speed support coefficient investigation reveals that urban green production has benefited from introducing the high-speed network over time. The most significant and central towns have seen a more significant increase in this trend, whereas the smallest and most eastern cities have decreased. The impact of high-speed rail opening on green TFP varies based on the city and region. The distinction lies in the magnitude of the stimulating effect on overall green urea production and the rate of its progressive growth.

## 5 Conclusion and policy implications

Even though the term “sustainable economic resurgence” is still mainly in the realm of theory, governments across the globe are taking action in response to the COVID-19 pandemic by investing in infrastructure and energy production that heavily relies on fossil fuels. Sustainable management of resources and environment management hinges on implementing effective tax policies. This scholarly piece demonstrates how the green sector has experienced a resurgence due to the Chinese government's reductions in energy-efficient enterprises. Our study utilized the Differential-In-Differences (DID) method to quantify the efficacy of these policies, analyzing quarterly data obtained from publicly traded Chinese companies. Our research findings conclude that embracing innovation is optimal for companies seeking to thrive in the post-COVID-19 green economic upswing. Businesses that reduce their energy consumption while maintaining their profit margins will likely reap greater rewards from their inventive strategies during challenging economic times. This could be attributed to the improved economic performance of these companies resulting from tax cuts, and this encourages them to support creativity and invest in novel goods, especially regarding their protection, availability, and corporate financing. Reducing the administrative and financial burdens on energy-efficient businesses can bolster their financial health, fostering an environment conducive to innovation. Additionally, as the efficiency of these enterprises increases, leading companies are increasingly willing to take the necessary risks to drive innovation. Further, by analyzing information regarding the intensity of the COVID-19 epidemic, we demonstrate that economic incentives significantly boost the innovative activities of energy-efficient enterprises.

Additionally, the progress in accessibility resulting from transportation investments is projected to contribute to regional economic development. The China Entrepreneurship Development Initiative set up a scientific methodology to investigate how improved delivery mechanisms affect urban economies. Creating businesses in China has continually improved the country's total green factor output, as the standard econometric findings show. Standard regression outcomes are credible because the critical explanatory variables have favorable regress values that survive stringent resilience and endogenous variables analysis. The experimental results indicate that the high-speed line can positively impact the city's overall green factor production by fostering the growth of green finance. The positive effects tend to peak early and gradually diminish. What's more, an organization's size and location will have a role in how much of an effect a business transformation program has on the development of sustainable financing.

### 5.1 Policy implications

I. The publishing is a gentle reminder that tax strategies focusing on ecological consciousness are instrumental in promoting economic growth after the pandemic and transitioning towards sustainable development. As a first step, our results suggest that company leaders include environmentally friendly liquidation managing approaches in impetus strategies, which has two significant ramifications for legislators. Authorities should think about how to effectively and quickly back initiatives that will hasten the transition to a sustainable economy. Prudent allocation of resources plays a crucial role in enabling effective operations and the successful management of innovations. Additionally, green tax policies create a level playing field for companies that enhance energy efficiency by facilitating information sharing. A carefully devised fiscal strategy has the potential to enhance market expectations by reducing the cost of external finance. Hence, officials and investors should exercise vigilance when formulating and enforcing green legislation.
II. The outcomes offer a glimpse into the blossoming urban economy of China, an aspect that holds great significance considering the nascent stage of enterprise innovation initiatives. Furthermore, the findings of this study serve as a valuable resource for governments and small businesses in emerging and developing nations as they make their way through the complex world of green financing and learn to reap the financial benefits of rapid transit. After COVID-19 arrived, a phase of epidemic emergence followed. To counterbalance the adverse impact of the pandemic on investor expectations, the government must establish a system that ensures environmentally friendly credit. Encouragement will be provided for investments in businesses that span borders and rely on transportation. In contrast, the ecological consequences of such investments will be mitigated through heightened bank involvement in green financing and the utilization of tax revenues as a protective measure. Compulsory. Additionally, the government can effectively mobilize substantial amounts of capital for green projects or companies beyond national borders, thereby diminishing the credit risk faced by commercial banks and facilitating the advancement of external green infrastructure. There are a total of nineteen distinct settings at one's disposal.
III. Here are a few ways this publication enhances prior research: Firstly, we employ socioeconomic concepts to construct a conceptual framework for the green business innovation system. Subsequently, we explore the mechanisms through which different policies impact the five subsystems mentioned earlier, thereby influencing the development of green business innovation. Furthermore, we develop a simulation to illustrate the impact of potential policy changes on these subsystems and, consequently, shape the corporate green innovation system. Lastly, we use a simulation to assess inventiveness in the Chinese sustainable industrial sector and provide a comprehensive evaluation of the necessary governmental implications encompassing fiscal, financial, technical, and environmental aspects. This ultimately establishes a favorable policy environment that fosters the advancement of innovative green technologies.

### 5.2 Limitation of study

The study investigative the effects of fiscal policies and green financing on firm novelty and firm value in the background of a green economic recovery recognizes a number of limitations that should be taken into account. It is important to note that the research's use of quarterly data from Chinese companies between Q1 2019 and Q2 2021. In addition, the preferred methodology of using a variance-variance analysis may not fully capture the complex connections between fiscal policies, green financing, and their impact on innovation and firm value. This approach might overlook non-linear relationships and the various factors that affect firm performance. The study's narrow emphasis on tax incentives and energy efficiency as catalysts for innovation and firm value also presents a drawback, as it may overlook other crucial factors like regulatory shifts, market demand for sustainable products, technological progress, and the influence of international collaboration in green technology industries. This limited perspective may delay the knowledge of a extensive range of elements that contribute to the recovery of the green economy. In addition, the analysis mainly focuses on the immediate impacts of these policies and mechanisms, neglecting to delve into the long-term practicality and development of these dynamics. Finally, the study's conclusion about the limited impact of its findings raises doubts about the overall effectiveness of eco-design fiscal policies as part of a comprehensive business transformation strategy for the post-COVID-19 era. This emphasizes an important area for further research, indicating the necessity of conducting a thorough examination into the circumstances that can best facilitate the contribution of fiscal policies and green financing to maintainable fiscal and firm growth. By expanding geographical coverage, conducting longitudinal studies, and increase the investigative scope, a more complete considerate can be gained regarding the ways fiscal policies and green financing drive innovation, enhance firm value, and support long-term green economic recovery.

## Ethics approval and consent to participate

Not applicable.

## Consent for publication

All of the authors consented to publish this manuscript.

## Funding

Funding information is not available.

## Data availability

We collected relevant data from World Bank open data available at https://data.worldbank.org/. For any further query on data, corresponding author at email address longtengfei1984@163.com may be approached.

## CRediT authorship contribution statement

**Long Tengfei:** Writing – review & editing, Writing – original draft, Methodology, Conceptualization. **Ahsaan Ullah:** Methodology, Data curation, Conceptualization.

## Declaration of competing interest

The authors declare that they have no known competing financial interests or personal relationships that could have appeared to influence the work reported in this paper.
